# Feasibility and Acceptability of Photographic Food Record, Food Diary and Weighed Food Record in People with Serious Mental Illness

**DOI:** 10.3390/nu13082862

**Published:** 2021-08-20

**Authors:** Annabel Sandra Mueller-Stierlin, Scott B. Teasdale, Uemmueguelsuem Dinc, Sabrina Moerkl, Nicole Prinz, Thomas Becker, Reinhold Kilian

**Affiliations:** 1 Department of Psychiatry II, University of Ulm, Lindenallee 2, 89312 Günzburg, Germany; Uemmueguelsuem.Dinc@Uni-Ulm.de (U.D.); T.Becker@Uni-Ulm.de (T.B.); Reinhold.Kilian@Uni-Ulm.de (R.K.); 2 School of Psychiatry, UNSW, Sydney, NSW 2052, Australia; scott.teasdale@unswalumni.com; 3 Universitätsklinik für Psychiatrie und Psychotherapeutische Medizin, Medizinische Universität Graz, 8036 Graz, Austria; sabrina.moerkl@medunigraz.at; 4 Institute of Epidemiology and Medical Biometry, ZIBMT, University of Ulm, 89081 Ulm, Germany; Nicole.Prinz@Uni-Ulm.de; 5 German Center for Diabetes Research (DZD), Munich-Neuherberg, 85764 Neuherberg, Germany

**Keywords:** diet, nutrition, mental illness, mental disorder, assessment

## Abstract

People with serious mental illness (SMI) experience challenges that may make typical dietary assessment methods less feasible and accurate. This study aims to determine the feasibility, acceptability and preliminary validity of a 3-day photographic food record (PR), a 1-day food diary (FD) and a 1-day weighed food protocol (WR) in people with SMI. Participants completed measures at two timepoints, with a 4-week interval. Feasibility and acceptability for each method were measured through four outcomes: percent of completers, quality assessment, number of participants requiring technical devices and satisfaction questionnaire. Relative validity was measured by agreement in estimated energy intake between methods, using Bland–Altman analysis and WR as the benchmark, and prevalence of misreporting, using the Goldberg cut-off method, updated by Black. In total, 63 participants were recruited, with a dropout rate of 19.0% prior to timepoint 1 and additional 6.4% prior to timepoint 2. Quality deficits were identified for all methods. The FD was most acceptable to participants, followed by the PR. The difference in estimated energy intake between assessment methods was not statistically significant, though there was considerable individual variability. Underreporting was considerable across all methods but appeared highest in the PR. A FD and PR present as feasible and acceptable methods for assessing dietary intake in people with SMI. Further validity testing is required. In addition, clear guidance for completion and removal of potential barriers is required for participants.

## 1. Introduction

People with severe mental illness have a 15 years shorter life expectancy than the general population [1]. Obesity and associated metabolic complications are important risk factors leading to cardiovascular diseases and other conditions [2,3]. Poor dietary habits together with other lifestyle factors such as lack of physical exercise, smoking and drug use have been identified as important behavioral elements that interactively enforce the negative side effects of modern psychoactive regimens [4].

Weight management strategies, including nutrition interventions, are considered a core component in clinical practice for managing the lasting physical health inequalities experienced by people living with mental disorders [5]. Methods for the assessment of energy imbalances are crucial requirements for the development and the evaluation of nutrition intervention programs.

However, despite a wide spectrum of dietary assessment methods have been developed, the feasibility, acceptability and validity of varying dietary assessment methods for specific use in people with severe mental illness (SMI) are unknown to date [6]. Dietary assessment methods should be accurate and feasible for those conducting the assessments, and they should be acceptable to people living with mental illness. Effective dietary assessment methods should also be sensitive to dietary changes over time in order to provide a solid basis for intervention evaluation.

Severe mental disorders, including schizophrenia, bipolar disorder and major depression, have in common that their clinical characteristics and their effects on daily living make the application of behavioral measures more difficult in the affected populations compared to physically ill or healthy people. These complications include impairments in cognition and functioning such as thought disorganization, poor memory and difficulty taking on new information [7,8,9]. This also includes a more unsteady lifestyle and a greater reluctance to provide information about daily habits. Frequently used dietary assessment methods require participant recall or maintaining a prospective record [6,10], both of which are potentially burdensome to participants with cognitive challenges. Innovative and user-friendly methods of measuring dietary intake for people living with mental disorders need to be investigated. Following the principle of “*what gets measured gets managed*”, an adequate estimation of dietary intake allows further targeted nutrition interventions which would not be possible otherwise.

Food-frequency questionnaires and brief dietary assessment instruments such as food diaries are low-cost and frequently used in people with SMI, each of which have varying degrees of burden to the participant [6]. More recently, developments in mobile technologies have given rise to image-based assessment. With this, all eating occasions are captured through handheld devices or wearable cameras for intake assessment by specialist clinicians such as dietitians [11]. This may present a more feasible and accurate method for people with SMI.

The aims of this study were, first, to assess the feasibility and acceptability of a 3-day photographic food record (PR), 1-day food diary (FD) and 1-day weighed food record (WR) among people with SMI, and, second, to gain first insight in the relative validity of PR, WR and FD when used by people with SMI.

## 2. Materials and Methods

### 2.1. Study Design

This feasibility, acceptability and validity study was conducted between 2016 and 2017. Ethical approval was obtained from Ulm University’s Ethics Commission, Germany (393/15). The study was registered with the German Clinical Trials Register (DRKS00010104) that is part of the International Clinical Trials Registry Platform and conducted in accordance with the Declaration of Helsinki, 2013. Written informed consent was obtained from all participants.

### 2.2. Participants

Participants were recruited via psychiatric clinics and socio-psychiatric service providers in Southern Germany, public media adverts and by contacting participants of previous studies. Inclusion criteria were: (i) aged between 18 and 65 years, (ii) self-reported SMI such as psychotic or affective disorder (ICD-10: F20 to F39), and (iii) inpatient or outpatient/community-dwelling. In the case of inpatients who consented to participate, data collection commenced post-hospital discharge. Exclusion criteria were: (i) primary diagnosis of a mental illness other than an SMI, (ii) potential participant reported that they are currently following a restrictive diet (e.g., for weight loss), and (iii) self-reported eating disorder.

### 2.3. Sample Size Calculation

A formal sample size calculation was not undertaken, as only descriptive analyses were conducted. According to comparable studies, 40 participants each with an affective disorder or a schizophrenia or related psychosis should be recruited [12,13].

### 2.4. Assessment Measures

Sociodemographic and medical history data were collected at baseline using a study-specific questionnaire. Data collected included: age, gender, education level, living situation, duration since the first onset of the SMI, number of admissions to a psychiatric inpatient unit, admission to a psychiatric inpatient unit within the last 12-months, current prescription of psychotropic medication, psychosocial impairment (Health of the Nation Outcomes Scale, HoNOS) [14,15,16,17], presence of a physical health condition, body mass index (BMI) [weight/height^2^], current smoking status and quality of life (WHO-QoL-BREF) [18].

Dietary assessment methods were:

- 3-day photographic diet record (PR);
- 1-day written food diary (FD);
- 1-day weighed food protocol (WR).

A study-specific satisfaction questionnaire was used to determine the practicability of the dietary assessment methods from the participants’ perspective. For this satisfaction questionnaire, there were six criteria for non-digital methods and nine criteria for the digital method, each of which required a response on a 4-point Likert scale for the degree to which participants agreed with the statement. Additionally, open-ended questions were included at the end of the survey to gain qualitative feedback on the measures. This satisfaction questionnaire has been used in previous studies evaluating the digital method [19].

A 9-item rating form was developed specifically for this study to assess data quality of the PR, and used by the investigator interpreting the images. Quality was recorded as high, moderate or low. The quality score for the 1-day FD was assessed as high or low by the investigator based on whether portion sizes were reported based on individual consumption rather than the standard serving sizes: if only standard serving sizes were checked for all foods listed (without individual adaptation), it was considered low quality. The quality score for the 1-day WR was assessed as high or low by the investigator based on whether individual ingredients and foods were listed and weighed (rather than just listing and weighing the final meal): if components for one meal or more were not listed and measured individually it was considered low quality.

### 2.5. Procedure

There were two assessment periods separated by a 4-week interval, each assessment period running over three consecutive days: Thursday to Saturday. Participants were randomly assigned to one of two groups (A, B) to complete the 1-day FD and the 1-day WR in opposing order, in a cross-over design. In both groups, the PR was conducted over the 3-days of both assessment timepoints. A satisfaction survey was also completed on each of the assessment days, with Thursdays and Fridays referring to the corresponding written record completed that day (either the FD or the WR), and the Saturdays referring to the PR. The flow of dietary assessment measure completion is described in Table 1.

At baseline, participants received both verbal and written instruction by a study investigator on the following procedures for each dietary assessment method:

A 3-day photographic diet record (PR): Participants were asked to take pictures in standing position, from the front of the plate at an angle of approximately 45° with a smartphone or digital camera (digital camera provided to participants by investigators where necessary). A reference object (2 euro coin) was to be placed next to the plate/bowl/food item for each photo to allow portion size estimation. Leftovers and extra food portions were to be documented by additional pictures, with an additional 2 euro coin to assist portion size estimation.

A booklet of varying food images with the exact weight known was used to estimate portion sizes. This booklet was first developed for the Second German National Nutrition Survey (NVS-II) [20], based on the EPIC-Soft picture book that was developed for the calibration study within the EPIC (European Prospective Investigation into Cancer and Nutrition) project supervised by the International Agency for Research on Cancer [21]. An expanded version was used for a previous photo-based diet record validation study in healthy adults [19].

A 1-day FD (FD): Participants were asked to record each individual food and beverage consumed over a 24-h period (12:00 am and 11:59 pm) in a guided FD. The guided FD lists the weight/volume of standard serving sizes for 127 food items and 25 beverages. In addition, there were three photographic illustrations (small, medium and large portion size) and accompany weight estimations for seven foods to assist in portion size estimation.

A 1-day weighed food record (WR): Participants were asked to weigh and record individually each food and beverage consumed between 12:00 am and 11:59 pm, including individual ingredients making up a meal weighed individually—using a conventional digital kitchen scale (provided to participants by investigators where necessary). The name of the food item (providing as much detail as possible), estimation of quantity and preparation method were documented.

Energy intake for all dietary measures was estimated using DGExpert software, version 1.9.3, developed by the German Nutrition Society.

### 2.6. Outcomes

Feasibility and acceptability of dietary assessment methods served as primary outcomes:

Feasibility: (i) percent of completers for each dietary assessment measure, (ii) data quality measured by the quality assessment questionnaires completed by the investigator, and (iii) number of participants that required a digital camera and/or digital kitchen scale from the investigator to perform the task.

Acceptability: measured by participants’ responses to the satisfaction questionnaire covering (i) six to nine items to be rated on a 4-point Likert scale, and (ii) qualitative, open-ended questions.

Relative validity and reliability of energy intake assessment were chosen as secondary outcomes:

Relative Validity and Reliability: (i) relative validity was measured through agreement of estimated energy intake on the same day between the PR methods and the WR, using WR as the benchmark, (ii) agreement of estimated energy intake between the FD and WR on consecutive days, using WR as the benchmark, (iii) agreement of energy intake on the same day between the PR and FD, (iv) prevalence of misreporting (under- and overreporting) at both the group level and individual level for all assessment methods, and (v) reliability was measured by testing the agreement between test/re-test values for estimated energy intake at timepoint 1 and timepoint 2.

### 2.7. Statistical Analysis

Descriptive statistics were used to explore the feasibility and acceptability of the three dietary assessment methods. Descriptive statistics included: mean and standard deviation or median and range, number of participants and percent of the sample. For descriptive purposes only, Fisher’s exact tests and paired *t*-tests for differences between timepoints and between dietary assessment methods were conducted for the satisfaction questionnaire.

For validity assessment, Spearman’s correlation analyses and the Bland-Altman-Method were used to assess the mean agreement for estimated energy intake between the methods and to test individual variability. A *p*-value less than 0.05 was chosen as threshold for statistical significance. Misreporting was explored for all three methods according to EFSA/EU Menu Guidance, based on the PILOT-PANEU project. The Goldberg cut-off method [22], updated by Black [23,24], was used to detect misreporting. Basal metabolic rate (BMR) was calculated using the Schofield equation [25], applying an adjustment of ×1.3 (low physical activity) given the lower physical activity level in people with SMI [26].

## 3. Results

### 3.1. Participants and Participants Flow

In total, 63 participants (Group A *n* = 31, Group B *n* = 32) with the following diagnoses consented to participate in the study: depressive disorder *n* = 36 (57%), bipolar disorder *n* = 4 (6%), schizophrenia and related psychoses *n* = 14 (22%) and other diagnoses *n* = 9 (14%), with mean illness duration of 14.4 (SD 12.0) years. Mean psychosocial impairment score (11.9) was within the threshold for SMI, cognitive problems (1.5) in average were rated as clinically minor (=1) or mild (=2). Eighteen participants (34%) had a psychiatric unit inpatient stay within the last 12 months, and 51 (86%) were currently prescribed psychotropic medication. Mean participant age was 45.7 (SD 11.8) years and 35 participants (58%) were female. The majority (58%) of participants completed secondary school or higher, about half (55%) of participants were single, and about one-third (37%) of participants lived alone and 15% of participants lived with minors. The mean BMI of 30.8 (SD 7.8) kg/m² was above the obesity threshold, and 60% of participants reported a comorbid physical health condition. Characteristics of the participants are presented in Table 2.

Of the 63 participants who consented to the study, 12 participants (19%, Group A *n* = 6, Group B *n* = 6) discontinued the study before or during the first assessment timepoint: affective disorder *n* = 7, psychotic disorder *n* = 4, and other *n* = 1, leaving 51 for analysis at timepoint 1. A further four participants (Group A *n* = 4, Group B *n* = 0) discontinued after the first assessment or during the second timepoint: affective disorder *n* = 3 and other *n* = 1, leaving 47 for analysis at timepoint 2. Reasons for dropout were: (i) burden of documentation, (ii) burden of the illness and (iii) employment commitments.

### 3.2. Primary Outcome: Feasibility

The number and percent of participants who completed the individual dietary measures for individual timepoints based on the original 63 participants are presented in Appendix A Table A1. The completion rate was comparable for WR (T1: 79.4%, T2: 71.4%), FD (T1: 76.2%, T2: 69.8%) and PR (T1: 79.4%, T2: 73.0%). 

The numbers of participants who required a digital camera and/or digital kitchen scale from the research investigator to complete the measurements were 5 participants (8%) for the PR and 11 participants (18%) for digital kitchen scales. 

A total of 2301 images were recorded through the photo-based dietary method, of which 82 (3.6%) were not relevant to the study. A mean 8.4 (SD 4.2) and 7.9 (SD 4.4) images were recorded per participant during assessment timepoints 1 and 2, respectively. The quality scores for PR are presented in Appendix A Table A2. High quality was met by ≥50% of the sample for six of the nine criteria in both timepoints, and by ≥75% of the sample for only two of the nine criteria. Considerable issues in quality were found for: (i) specific ingredients being identifiable, (ii) image quality and (iii) completeness when compared to FD and weighed protocol.

For 19.4% of FD, participants used the standard serving size only and did not adjust for the individual portion size. For 43.2% of WR, only the complete meal was weighed and not the individual ingredients.

### 3.3. Primary Outcome: Acceptability

Responses to the satisfaction survey are presented in Table 3 and in Appendix A Table A3. Participant satisfaction seems higher for the PR and the FD, when compared to the WR. This is equivalent to the participants’ preferred method: (i) FD (moderate–high preference level), (ii) PR (moderate–high preference level) and (iii) WR (low preference level). For amount of time and whether the method can be applied away from home, there was moderate–high satisfaction for PR, moderate satisfaction for FD and low satisfaction for the WR. There was moderate agreement for applying both the FD and PR over a longer assessment period, compared to low agreement for the WR.

Qualitative data reveal that participants judged the FD as the simplest/best followed by the PR. No participant considered the WR as the simplest/best and participants frequently reported this method as difficult. Some participants also acknowledged that data collection was incomplete for WR and PR. Reasons for incomplete data collection were: (i) participants forgot to weigh the food or take a picture, (ii) did not have the required equipment at hand or (iii) did not want to do it with others nearby. These problems are especially prevalent when eating away from home. Five participants acknowledged that they under-ate during the assessment period as a result of recording their consumption.

### 3.4. Secondary Outcomes: Relative Validity and Reliability

According to the lower Goldberg cut-off at group level, the majority of the participants have under-reported their energy intake; with two exceptions: The mean ratio for estimated energy intake to BMR (EEI:BMR) was plausible for the WR at timepoint 1, and above the upper cut-off for FD at timepoint 1.

At individual level, plausible intakes were found in 82% (both timepoints) of participants for the 1-day WR, and in 81% (timepoint 1) and 75% (timepoint 2) of participants for the 1-day FD. For the 3-days of PR, plausible intakes were found in 64% (timepoint 1) and 65% (timepoint 2). When using the PR on individual days, rate of plausible intakes increased to up to 74%.

Probably under-reporting was more frequent than over-reporting. The prevalence of under-reporting ranged from 13% to 18% for WR, 8% to 25% for 1-day FD and 23% to 33% for the PR (based on individual days). Further data on misreporting is presented in Table 4.

The mean energy intake was estimated between 1689 kcal/day (PR at the day of the WR at timepoint 1) and 2415 kcal/day (FD at timepoint 1) with standard deviations for 1-day assessment ranging from 800 (PR at the day of the WR at timepoint 1) to 1191 (FD at timepoint 1). The repeatability coefficient over 4 weeks ranges from 1701 kcal (PR) to 2439 kcal (FD). Further data on estimated energy intake and repeatability coefficients are provided in Table 5 and Table 6.

Estimated energy intake seems lower for the PR compared to the WR (mean difference: −325 kcal and −150 kcal for the two timepoints) and the FD (mean difference: −632 kcal and −191 kcal for the two timepoints). Estimated energy intake seems lower for the WR compared to the FD at timepoint 1 (mean = −449 kcal), but not at timepoint 2 (mean = 35 kcal). These differences were not statistically significant. The limits of agreement between methods are within the repeatability coefficients. The agreement of the measurements within individual assessment methods is comparable to the agreement of the measurements between assessment methods. Further data on mean energy intake differences and Spearman’s Correlation Coefficients between methods are provided in Table 7 and Table 8.

## 4. Discussion

To the authors’ knowledge, this is the first study to examine the feasibility, acceptability, reliability and relative validity of dietary assessment in people with SMI [6]. We found that the FD and PR methods were more feasible and acceptable than the WR method among people living with SMI. There was wide variability for the estimated energy intake for the FD, PR and the benchmark (WR). Moreover, there was considerable intra-individual variability of estimated energy intake between the two assessment timepoints. Hence, differences between two timepoints or between different methods were up to two to three times the daily energy requirement. The agreement of the measurements within individual assessment methods was comparable to the agreement of the measurements between assessment methods.

In line with other studies [19,27], PR and FD seemed more feasible and acceptable than WR, with the weighing of individual ingredients and foods considered burdensome. Completion rates for the dietary assessment methods ranged from 70% to 80%.

However, recruitment challenges should also be considered and may reflect a lower willingness and ability to record dietary intake overall in comparison to the sample who participated.

Despite the potential for financial, living and educational barriers, a small proportion of participants lacked the necessary equipment for the WR or PR methods. However, barriers were reported in using the camera and the kitchen scales when outside the home, and there were reports of self-perceived stigma when using the equipment in other people’s presence. These barriers might have led to under-reporting.

The high rate of energy intake under-reporting reduces confidence in the reliability for all three methods in this population group. However, they are comparable to rates in the general population: 11–50% in the general population, with the frequency of underreporting higher in people with BMI ≥ 30 kg/m^2^ [28]. These issues may impede nutritional psychiatry research progress and highlight challenges for dietary assessment in routine clinical care where nutrition intervention is recommended as part of multi-modal health promotion programs in psychiatric treatment guidelines [29].

Similar to a study with healthy volunteers [19], we found that the reliability and relative validity of the PR method seems not to differ from conventional methods (WR and FD) in people with SMI. However, due to the wide variability of differences in estimated energy intake between measures, the estimated energy intakes are likely not to be of practical relevance. Moreover, validity findings in people with SMI reported in this study are preliminary. Further studies are required and should use ‘gold standard’ techniques as benchmarks, e.g., doubly labeled water instead of the WR which lacks evidence of validity in this target population. In addition, nutrient-specific validity testing using biomarkers (e.g., carotenoids) is needed for future research on the association of macro- and micronutrients intake and physical and mental health outcomes.

Due to substantial heterogeneity in dietary assessment methods and study design, it is not possible to draw firm conclusions on the validity of these methods in people with SMI compared to that found in the general population. It is conceivable that the accuracy and reliability of these dietary assessment methods may be lower than in the general population due to perceptual and non-social cognitive deficits in people with SMI [30]. As mental disorders are heterogeneous in terms of psychosocial, functional and cognitive impairments, sample sizes of future studies should account for subgroup analysis by diagnostic group and control groups without mental illness should be included. In this study, subgroup analysis by diagnostic criteria was not possible due to the limited number of participants diagnosed with a psychotic disorder. Despite recruitment efforts, we were not able to overcome common challenges in recruiting people with psychotic disorders [31]. It is unclear to what degree a lower willingness to record dietary data and the complex study design contributed to the low recruitment rates. The dropout rate for people who consented to participate in this study (25%) is similar to the dropout rate derived from a meta-analysis of dropout rates from physical activity interventions in people with schizophrenia (26.7%) [32].

A further consideration is that the features of schizophrenia spectrum disorders and affective disorders change over time [33], and correspondingly, the medication and the functional impairment are likely to change as well [34]. Any changes in the occurrence of, for example, medication-related food craving [35,36], food insecurity [37] and motivation: loss of drive to shop and cook [38], also influence dietary behavior. A one-time recording is thus only a snapshot and the high repeatability coefficient may only reflect the actual variability in dietary intake of people with mental illness over time.

In terms of the feasibility of the method in everyday clinical practice, it should be considered that each of the dietary assessment methods employed in this study required considerable interpretation and analysis skills on behalf of the investigator. Recent studies revealed that image-based dietary assessment is challenging for dietetics students, interns and nutrition professionals and conclude that food-based serving size training is recommended to improve quantification accuracy [39,40]. Future studies in SMI population groups should test inter-rater reliability of varying measures both when interpreted and analyzed by expert clinicians such as dietitians, and when interpreted and analyzed by non-experts (e.g., mental health workers). This will guide requirements for dietary assessment research, training and implementation in mental health settings. Regardless of whether expert clinicians are involved in the data interpretation and analysis, comprehensive protocols for completing each dietary assessment method appear necessary to combat the cognitive and other challenges experienced by people with SMI.

## 5. Conclusions

One-day FD and PR assessments were found to be feasible and acceptable methods to assess dietary intake among people with SMI, whereas a WR was not. Regardless of the method chosen, clear guidance is required with respect to potential barriers such as lack of a digital camera. Due to the high individual variability observed, it is not possible to draw conclusions about the agreement of estimated energy intake when using PR or FD against WR. Further criterion validity assessments are required against ‘gold-standard’ measures such as the doubly labelled water technique and biomarkers. Ways to minimize under-reporting must be explored.

## Figures and Tables

**Table 1:** Schedule for completion of dietary assessment and satisfaction measures.

Group	Assessment Measure	1st Assessment Timepoint			2nd Assessment Timepoint		
		Thursday	Friday	Saturday	Thursday	Friday	Saturday
Group A	PR	X	X	X	X	X	X
	FD	X				X	
	WR		X		X		
	Satisfaction questionnaire	X (FD)	X (WR)	X (PR)	X (WR)	X (FD)	X (PR)
	Open-ended questions	X (FD)	X (WR)	X (PR)	X (WR)	X (FD)	X (PR)
Group B	PR	X	X	X	X	X	X
	FD		X		X		
	WR	X				X	
	Satisfaction questionnaire	X (WR)	X (FD)	X (PR)	X (FD)	X (WR)	X (PR)
	Open-ended questions	X (WR)	X (FD)	X (PR)	X (FD)	X (WR)	X (PR)

FD: Food diary, PR: Photographic record, WR: Weighed record.

**Table 2:** Characteristics of 63 participants who consented to participate in the study.

Participant Characteristic	Proportion or Mean Score
Age in years (M ± SD)	45.7 ± 11.8
Female (*n*, %)	35 (58.3%)
Education	
Secondary school or higher (*n*, %)	35 (58.3%)
University degree (*n*, %)	5 (8.5%)
Being single	33 (55.0%)
Living situation	
Living alone	22 (37.3%)
Living with minors	9 (15.3%)
Diagnosis	
Affective disorder (*n*, %)	40 (63.5%)
Psychotic disorder (*n*, %)	14 (22.2%)
Other mental illness (*n*, %)	9 (14.3%)
Duration of illness in years (m ± SD)	14.4 ± 12.0
Number of inpatient stays in psychiatric clinics (M, min-max)	2 (0–39)
Psychiatric inpatient stay in the last 12 months (*n*, %)	18 (34%)
Prescribed psychotropic medication (*n*, %)	51 (86.4%)
HoNOS total (m ± SD)	11.9 ± 5.6
HoNOS cognitive problems (m ± SD)	1.5 ± 0.9
Comorbid physical illness (*n*, %)	36 (60.0%)
BMI (m ± SD)	30.8 ± 7.8
Current smoker (*n*, %)	26 (43.3%)
QoL-Bref (m ± SD)	48.5 ± 25.0

BMI: Body mass index, HoNOS: Health of the Nation Outcome Scales, m: mean, M: Median, *n*: number of participants, QoL-Bref: World Health Organisation Quality of Life-Bref, SD: Standard deviation.

**Table 3:** Absolute and relative frequencies of participants who (rather) agree to the questions in the satisfaction survey for the different assessment methods.

*n* (%) ^1^	PR		FD		WR	
	Timepoint 1	Timepoint 2	Timepoint 1	Timepoint 2	Timepoint 1	Timepoint 2
In general, I was satisfied with the METHOD to assess dietary intake.	42 (88%)	37 (82%) *	44 (92%)	36 (78%) *	21 (46%)	24 (55%) *
The method is user-friendly.	43 (90%)	36 (80%) *	42 (88%)	38 (83%)	25 (53%)	18 (40%) *
The method requires a low amount of time and can be completed away from home.	34 (72%)	32 (74%) *	31 (65%)	25 (57%) ^#^	6 (13%)	6 (14%) *
The method has no effect on my choice of food.	39 (83%)	39 (89%)	39 (81%) ^#^	40 (87%) *	39 (83%) ^#,+^	34 (79%)^+^
I would prefer this method over other methods.	27 (63%)	31 (74%) *	36 (80%)	29 (66%) *	9 (21%)^+^	9 (21%)*
I can imagine applying this method over a longer period (e.g., 1 month).	26 (57%)	25 (61%) *	31 (66%) ^#^	24 (55%) *	9 (20%) ^#^	9 (21%) *
I would like daily feedback on my diet.	23 (48%)	26 (59%) *	N/A	N/A	N/A	N/A
The technical implementation caused no or small challenges.	39 (81%)	36 (80%)	N/A	N/A	N/A	N/A
An app for the PR would be useful.	31 (76%)	28 (68%) *	N/A	N/A	N/A	N/A

FD: Food diary, PR: Photographic food record, WR: Weighed food record. ^1^ *n* (%): absolute frequency (relative frequency) of participants who (rather) agree, * *p* < 0.05 for timepoint 1 vs. timepoint 2, # *p* < 0.05 for FD/WR vs. PR, + *p* < 0.05 for WR vs. FD.

**Table 4:** Rates of plausible and implausible intakes for each assessment method.

Time Point	Method	Sample (*n*)	Lowe Cut-Off	Upper Cut-Off	Mean (EI/BMR)	Under-Reporters		Plausible Reporters		Over-Reporters	
						*n*	%	*n*	%	*n*	%
T1	WR	50	1.20	1.41	1.20	9	18%	41	82%	0	0%
T2	WR	45	1.19	1.42	1.19	6	13%	37	82%	2	4%
T1	FD	48	1.20	1.41	1.45	4	8%	39	81%	5	10%
T2	FD	44	1.19	1.42	1.14	11	25%	33	75%	0	0%
T1	PR (day of WR)	50	1.20	1.41	1.02	13	26%	37	74%	0	0%
T2	PR (day of WR)	43	1.19	1.42	1.05	10	23%	32	74%	1	2%
T1	PR (day of FD)	49	1.20	1.41	1.05	13	27%	36	74%	0	0%
T2	PR (day of FD)	46	1.19	1.41	1.04	15	33%	30	65%	1	2%
T1	PR (3 days)	50	1.22	1.38	1.04	17	34%	32	64%	1	2%
T2	PR (3 days)	46	1.22	1.39	1.01	16	35%	30	65%	0	0%

BMR: Basal metabolic rate, EI: Estimated energy intake, FD: 1-day Food diary, PR: Photographic food record, *n*: number of participants, WR: 1-day Weighed food record.

**Table 5:** Mean energy intake (kcal/day) using different dietary assessment methods at timepoint 1 and 2.

Measures	Timepoint	*n*	Mean	SD	Min	Max
WR	1	50	1984 ^#,+^	818	454	3847
	2	45	1954	931	206	5289
FD	1	48	2415 ^#^	1191	726	6839
	2	44	1886 *	903	233	4172
PR (WR day)	1	50	1689	800	374	4135
	2	43	1748	863	102	4040
PR (FD day)	1	49	1770	828	392	4012
	2	46	1747	946	176	5413
PR (3 days)	1	50	1728	664	597	3553
	2	46	1693	727	421	3865

FD: Food diary, PR: Photographic food record, WR: Weighed food record * *p* < 0.05 for timepoint 1 vs. timepoint 2, # *p* < 0.05 for FD/WR vs. PR, + *p* < 0.05 for WR vs. FD.

**Table 6:** Repeatability coefficient data for between assessment points difference in energy intake (kcal/day).

Method		N	Mean	SD	Min	Max	Repeatability Coefficient
WR	T1–T2	44	15	1072	−3772	2321	±2101
FD	T1–T2	43	545	1244	−1423	5308	±2439
PR (WR day)	T1–T2	43	−61	1080	−3666	2409	±2117
PR (FD day)	T1–T2	45	72	868	−2019	1717	±1701
PR (3 days)	T1–T2	46	52	664	−1496	1478	±1302

FD: Food diary, PR: Photographic food record, WR: Weighed food record.

**Table 7:** Differences in estimated energy intake (kcal/day) between measures.

Measures	Timepoint	*n*	Mean	SD	Min	Max	95% CI	
WR and PR	1	49	325	854	−1734	2433	−1347	1998
	2	41	150	722	−1804	2035	−1265	1564
WR and FD	1	47	−449	1164	−4242	2100	−2731	1833
	2	44	35	1005	−2633	3614	−1934	2004
FD and PR	1	47	632	1177	−1390	6104	−1674	2938
	2	43	191	886	−1652	2198	−1546	1928

FD: Food diary, PR: Photographic food record, WR: Weighed food record. The PR day included in the analyses coincided with the day of the other measure.

**Table 8:** Spearman’s correlation coefficients between measures.

Timepoint 1	PR (3 Days)	PR (FD Day)	FD	PR (WR Day)	WR
PR (3 days)	1				
PR (FD day)	0.708 **	1			
FD	0.600 **	0.571 **	1		
PR (WR day)	0.813 **	0.429 **	0.353 *	1	
WR	0.537 **	0.408 **	0.486 **	0.411 **	1
**Timepoint 2**	**PR (3 Days)**	**PR (FD Day)**	**FD**	**PR (WR Day)**	**WR**
PR (3 days)	1				
PR (FD day)	0.856 **	1			
FD	0.504 **	0.441 **	1		
PR (WR day)	0.878 **	0.705 **	0.603 **	1	
WR	0.671 **	0.663 **	0.532 **	0.697 **	1

* correlation is significant at the 0.05 level (2-tailed), ** correlation is significant at the 0.01 level (2-tailed). FD: Food diary, PR: Photographic food record, WR: Weighed food record.

## Data Availability

The data presented in this study are available on request from the corresponding author. The data are not publicly available due to the used data protection declaration. The source data on dietary intake (food diaries, photographic food records and weighed food records) must not be shared.

## Appendix A

**Table A1:** Number and percent of participants who completed the different assessment methods.

Method	Timepoint	Number Completed	Completion Rate
WR	1	50	79.4%
	2	45	71.4%
FD	1	48	76.2%
	2	44	69.8%
PR (WR day)	1	50	79.4%
	2	43	68.3%
PR (FD day)	1	49	77.8%
	2	46	73.0%
PR (3 days)	1	50	79.4%
	2	46	73.0%

FD: Food diary, PR: Photographic food record, WR: Weighed food record.

**Table A2:** Quality scoring for the PR method assessed by a research analyst.

Item	1st Assessment Timepoint (*n*, %)			2nd Assessment Timepoint (*n*, %)		
	High	Moderate	Low	High	Moderate	Low
Use of coin	33 (65%)	4 (8%)	14 (27%)	28 (60%)	4 (9%)	15 (32%)
Realistic portion size	27 (53%)	16 (31%)	8 (16%)	26 (55%)	15 (32%)	6 (13%)
Specific foods identifiable	32 (63%)	17 (33%)	2 (4%)	36 (77%)	10 (21%)	1 (2%)
Specific Ingredients identifiable	15 (29%)	30 (59%)	6 (12%)	18 (38%)	20 (43%)	9 (19%)
Portion size could be estimated	39 (77%)	11 (22%)	1 (2%)	27 (57%)	16 (34%)	4 (9%)
Certainty to whether portion shown corresponds to intake	46 (90%)	2 (4%)	3 (6%)	36 (77%)	8 (17%)	3 (6%)
Image quality	21 (41%)	20 (39%)	10 (20%)	13 (28%)	25 (53%)	9 (19%)
Clear assignment to assessment day	32 (63%)	13 (26%)	6 (12%)	32 (68%)	8 (17%)	7 (15%)
Completeness in comparison to FD & WR	20 (41%)	20 (41%)	9 (18%)	22 (49%)	17 (38%)	6 (13%)

*n*: number of participants.

**Table A3:** Participant responses in the satisfaction survey for the different assessment methods.

m (SD) ^1^	PR		FD		WR	
	Timepoint 1	Timepoint 2	Timepoint 1	Timepoint 2	Timepoint 1	Timepoint 2
In general, I was satisfied with the METHOD to assess dietary intake.	2.4 (0.76)	2.2 (0.94)	2.4 (0.65)	2.2 (0.89)	1.5 (1.13)	1.6 (1.08)
The method is user-friendly.	2.3 (0.78)	2.2 (0.90)	2.3 (0.74)	2.2 (0.89)	1.6 (1.09)	1.2 (1.07)
The method requires a low amount of time and can be completed away from home.	2.0 (0.92)	2.0 (0.97)	1.9 (1.00)	1.8 (1.01)	0.6 (0.83)	0.7 (0.88)
The method has no effect on my choice of food.	2.4 (1.01)	2.6 (0.82)	2.4 (0.94)	2.5 (0.89)	2.4 (0.93)	2.3 (1.05)
I would prefer this method over other methods.	1.8 (1.01)	2.0 (1.02)	2.1 (0.91)	1.9 (1.06)	0.8 (0.98)	0.8 (1.04)
I can imagine applying this method over a longer period (e.g., one-month).	1.7 (1.09)	1.9 (1.11)	1.9 (1.13)	1.7 (1.10)	0.7 (1.03)	0.8 (1.04)
I would like daily feedback on my diet.	1.6 (1.09)	1.8 (1.13)	N/A	N/A	N/A	N/A
The technical implementation caused no or small challenges.	2.1 (0.87)	2.1 (0.99)	N/A	N/A	N/A	N/A
An app for the PR would be useful.	2.2 (0.97)	2.1 (1.10)	N/A	N/A	N/A	N/A

m (SD) ^1^: mean (standard deviation).
